# Is It Possible to Predict the Presence of Intestinal Angioectasias?

**DOI:** 10.1155/2014/461602

**Published:** 2014-03-17

**Authors:** Tiago Cúrdia Gonçalves, Joana Magalhães, Pedro Boal Carvalho, Maria João Moreira, Bruno Rosa, José Cotter

**Affiliations:** ^1^Gastroenterology Department, Centro Hospitalar do Alto Ave, 4831-044 Guimarães, Portugal; ^2^Life and Health Sciences Research Institute, University of Minho, 4710-057 Braga/Guimarães, Portugal; ^3^ICVS/3B's, PT Government Associate Laboratory, 4710-057 Braga/Guimarães, Portugal

## Abstract

*Background and Aim*. Angioectasias are the most common vascular anomalies found in the gastrointestinal tract. In small bowel (SB), they can cause obscure gastrointestinal bleeding (OGIB) and in this setting, small bowel capsule endoscopy (SBCE) is an important diagnostic tool. This study aimed to identify predictive factors for the presence of SB angioectasias, detected by SBCE. *Methods*. We retrospectively analyzed the results of 284 consecutive SBCE procedures between April 2006 and December 2012, whose indication was OGIB, of which 47 cases with SB angioectasias and 53 controls without vascular lesions were selected to enter the study. Demographic and clinical data were collected. *Results*. The mean age of subjects with angioectasias (70.9 ± 14.7) was significantly higher than in controls (53.1 ± 18.6; *P* < 0.001). The presence of SB angioectasias was significantly higher when the indication for the exam was overt OGIB versus occult OGIB (13/19 versus 34/81, *P* = 0.044). Hypertension and hypercholesterolemia were significantly associated with the presence of SB angioectasias (38/62 versus 9/38, *P* < 0.001 and 28/47 versus 19/53, *P* = 0.027, resp.). Other studied factors were not associated with small bowel angioectasias. *Conclusions*. In patients with OGIB, overt bleeding, older age, hypercholesterolemia, and hypertension are predictive of the presence of SB angioectasias detected by SBCE, which may be used to increase the diagnostic yield of the SBCE procedure and to reduce the proportion of nondiagnostic examinations.

## 1. Introduction

Obscure gastrointestinal bleeding (OGIB) remains one of the most challenging issues for gastroenterologists. It is defined as bleeding from gastrointestinal (GI) tract that persists or recurs without obvious etiology after esophagogastroduodenoscopy (EGD), colonoscopy, and radiologic evaluation of the small bowel (SB). OGIB accounts for about 5% of the cases of gastrointestinal bleeding [1]. The source of bleeding is located in the SB in about 75% of cases, where lesions can be scarcely accessed by standard endoscopy [1, 2]. These patients frequently require several blood transfusions and repeated hospital admissions, being at risk for complications not only related to anemia, but also caused by numerous exploratory procedures [3]. As a consequence, OGIB negatively affects patients' quality of life and represents a significant burden of healthcare resources [3].

Small bowel capsule endoscopy (SBCE) has become a particularly useful tool in the management of OGIB, which remains the most common indication for the procedure [4]. Several studies have confirmed the higher diagnostic yield of SBCE in diagnosing the cause of OGIB when compared to other investigative procedures [2, 5–10].

The causes of GI bleeding originating in the SB include tumours, chemical/radiation injuries, vascular diseases, inflammatory diseases, systemic diseases, or infectious diseases [3]. The prevalence of those types of lesions varies with patient's age; for instance, SB tumours (such as gastrointestinal stromal cell tumours, carcinoid tumours, adenocarcinomas, and lymphomas), Dieulafoy's lesion, inflammatory bowel disease, and Meckel's diverticulum are the most common causes at younger ages [1, 11, 12], while older patients are more likely to bleed from vascular lesions or nonsteroidal anti-inflammatory drug (NSAID) induced SB disease [1, 12]. Overall, angioectasias are the most common origin of OGIB, being responsible for approximately 30–40% of the cases [3, 13, 14]. Angioectasias consist of dilated, ectatic, tortuous, thin-walled vessels of the mucosa or submucosa, without inflammation or fibrosis [13, 15].

The clinical significance of angioectasias has not been fully elucidated, and the optimal approach to these lesions is still controversial, although it is reasonable to assume that it is important to detect angioectasias in order to plan medical or endoscopic treatment when indicated. However, despite being the most common lesions found during OGIB investigation, there are still a considerable number of cases in which neither angioectasias nor other potentially bleeding lesions can be found after a SBCE examination. Those cases of non-diagnostic SBCE represent increased costs and an undesirable expenditure of healthcare resources, which could theoretically be reduced if predictive factors of lesions such as angioectasias could be identified and used to properly select patients to undergo SBCE.

The aim of this study was to collect demographic and clinical data of patients who were investigated for OGIB with SBCE and analyze whether any of those factors could be used to predict the presence of SB angioectasias.

## 2. Methods

This study was designed as a single-center, retrospective cohort investigation. All patients referred to our center for investigation of OGIB with SBCE between April 2006 and December 2012 were reviewed. OGIB was defined according to the published medical position statement of the American Gastroenterological Association (AGA) [1]. This condition was further divided on occult OGIB (positive fecal occult blood test or iron deficient anemia) and overt OGIB (passage of visible blood) [1]. Demographic characteristics (namely, age, gender, and race) and relevant medical history (such as indication for SBCE, hypertension, diabetes mellitus, hypercholesterolemia, tobacco use, chronic kidney disease (CKD), chronic liver disease, aortic stenosis, previous abdominal surgery, antiplatelet, or anticoagulant drugs use) were obtained by consultation of patients' medical records.

### 2.1. SBCE Procedure

All the procedures were performed using PillCam SB or PillCam SB2 capsules from GIVEN Imaging (Yoqneam, Israel). Before the procedure, each patient received general instructions and informed consent was obtained. Patients had a clear liquid diet the day before capsule ingestion and an overnight 12-hour fast. Patients were allowed to drink fluids after 2 hours and to have a light meal after 4 hours. Consensual contraindications for SBCE procedure were respected and have been described elsewhere [16–18].

### 2.2. Analysis of SBCE Findings

The complete video obtained in each SBCE was reviewed by two gastroenterologists with vast experience in capsule endoscopy. Angioectasias were identified as flat to slightly elevated, clearly demarcated, bright red vascular lesions, with variable size.

### 2.3. Inclusion Criteria

Patients with OGIB submitted to SBCE during the evaluation of OGIB with at least one SB angioectasia were included. Those patients investigated with SBCE for the same reason and without any finding during the examination were also included and used as the control group.

### 2.4. Exclusion Criteria

To minimize bias, patients with vascular lesions of the SB other than angioectasias, such as phlebectasias, portal enteropathy, or SB varices, were not included in the study. Patients in which the capsule detected other potentially bleeding lesions such as ulcers, erosion, or tumours were also excluded. Other patients had an incomplete SBCE and were not considered eligible for this study.

### 2.5. Data Analysis

Statistical analysis was performed using the Statistical Packages for Social Sciences software (SPSS) version 17.0. Differences between groups were evaluated using Student's* t*-test for quantitative variables and *χ* ^2^ test for nominal variables. A* P* Value <0.05 was considered statistically significant.

## 3. Results

### 3.1. Population Description

From April 2006 to December 2012, a total of 284 patients underwent SBCE examination in our center for etiologic investigation of OGIB. Of those, 100 patients met the inclusion criteria and were selected to enter the study. All patients swallowed the capsule without difficulty and none had any complication related to the procedure. SBCE detected at least one SB angioectasia in 47 patients. On the other hand, 53 patients were found to have no abnormalities and were included in the control group.

### 3.2. Demographic Data

We found that the mean age in the control group was 53.1 ± 18.6 years (range, 28–87), while, in the angioectasia group, it was 70.9 ± 14.7 years (range, 19–91). This difference was found to be statistically significant (*P* < 0.001). As for gender, the control group was composed of 18 men (34%) and 35 women (66%), whereas the angioectasia group had 23 men (49%) and 24 women (51%). In contrast to what was found with age, there were no significant differences between genders (*P* = 0.156). Lastly, all patients included in our study were Caucasian, so comparison between different races and the presence of angioectasias was not possible.

### 3.3. Relevant Medical History Data

The indication for the SBCE was one of the studied points, so division of patients between those with occult OGIB and those with overt OGIB was done. Globally, 81 patients were submitted to SBCE for the study of occult OGIB and the other 19 performed the capsule examination because they had overt OGIB. When subdivided, we found out that, in the control group, 47 patients (89%) had occult OGIB and only 6 (11%) had overt OGIB. By contrast, in the angioectasia group, 34 patients (72%) presented with occult OGIB and 13 patients (28%) had overt OGIB. There was a statistic difference between the two groups (*P* = 0.044).

From the conditions considered relevant in patients' past medical history, special attention should be put towards hypertension and hypercholesterolemia. Regarding hypertension, 29 patients (55%) in the control group had normal blood pressure values and 24 patients (45%) had diagnosis of hypertension, while, in the angioectasia group, however, 38 patients (81%) had hypertension but only 9 patients (19%) were normotensive. This result reached statistical significance (*P* < 0.001). As far as hypercholesterolemia is concerned, 34 patients (64%) in the control group had a normal lipid panel and 19 (36%) had altered lipid values. On the opposite side, in the angioectasia group, 19 patients (40%) had no lipid changes while 28 patients (60%) had diagnosed hypercholesterolemia. This finding was also statistically significant (*P* = 0.027).

As mentioned previously, other conditions were searched in the studied patients. In this investigation, the diagnoses of diabetes mellitus, chronic kidney or liver diseases, aortic stenosis, as well as previous abdominal surgery, or tobacco use were not significantly different between the two groups. In addition, regarding medication use, namely, antiplatelet or anticoagulant drugs, no differences were found among the control or the angioectasia groups.

The results are summarized in Table 1.

## 4. Discussion

Angioectasias can be found throughout the entire GI tract. They may be clustered in one single region or they may coexist in several different GI locations. The pathogenesis of GI angioectasias is not fully understood, but different theories had already been proposed. Some authors advocate that angioectasias should be regarded as degenerative lesions of aging, caused by chronic intermittent low-grade obstruction of veins, capillaries, and arterioles that supply the mucosa [19]. Other authors defend the theory of a neurohormonal abnormality in which sympathetic nerves may stimulate intestinal vascular smooth muscle relaxation in response to hypoperfusion. Chronically, local vascular overload, dilation, and ectasia may pathologically develop leading to permanent angioectasia [13, 19]. Overexpression of angiogenic factors and deficient production of type IV collagen have also been suggested as pathogenic factors [20, 21].

In our study, we observed that age was significantly associated with higher prevalence of SB angioectasias, which is in line with the results of other previous studies, endorsing the possibility of a degenerative process behind the pathogenesis of GI angioectasias [13, 15, 22]. No significant differences were observed between the two groups regarding gender. It is frequently reported that the diagnostic yield of SBCE is the highest for patients with ongoing overt OGIB, although it has not been consistent in all studies [23, 24]. Indeed, the diagnostic yield of SBCE seems to be closely related to the timing of the procedure and it has been recommended that it should be performed early in the work-up of patients with OGIB [25, 26]. In our study, we found a significant association between overt OGIB and the presence of SB angioectasias. However, the exact time interval between the bleeding episode and the SBCE examination was not analyzed in our study.

We analyzed some major vascular disease risk factors, particularly, hypertension, diabetes mellitus, hypercholesterolemia, and smoking. Hypertension is a very common clinical finding in patients with GI angioectasias [15, 27, 28]. In our study, about 80% of patients with angioectasias had a known diagnosis of hypertension, being reasonable to admit that, similarly to what happens in other vascular territories (like in brain, eye, kidney, or heart microcirculations), hypertension might have a role in the aging of GI blood vessel, leading to the development of identifiable vascular lesion such as angioectasias. Diabetes mellitus can recognizably damage both endothelial and smooth muscle cells, which chronically cause degenerative alterations in blood vessels and impair tissue healing [29]. In our study, we found that the prevalence of diabetes mellitus in both control and angioectasia groups was similar. We also analyzed hypercholesterolemia as a possible risk factor for angioectasias, not only because of its prevalence in western populations but also because of that it plays as well an important role in the pathogenesis of vascular dysfunction [30]. Although some studies report no association [28], we found that more than an half of patients with SB angioectasias had hypercholesterolemia versus about one third of patients in the control group, which was statistically significant. Regarding smoking, it has been proven to have deleterious effects on the regulation of gastric microcirculation, consequently leading to some recognized causes of GI bleeding such peptic ulcer disease [31, 32]. While some studies report higher rates of tobacco use among patients with angioectasias, others could not support such an association [33, 34]. In our study, smoking was twice as common in the angioectasia group, although it did not reach statistical significance.

We also investigated some other conditions possibly associated with the presence of GI angioectasias, such as chronic kidney disease (CKD). Angioectasias have been reported to be a common cause of upper GI bleeding in patients with CKD [35] and some studies identified angioectasias as the leading cause of recurrent lower GI bleeding in those patients [36]. The reason for this association is still unknown, although a possible explanation is that angioectasias may not be more common but could bleed more easily due to uremia-induced platelet dysfunction. In our study, few patients with CKD were included and the differences between groups were not significant. It has also been suggested as an association between GI bleeding from angioectasias and aortic stenosis, also known as Heyde's syndrome [37]. In our study, very few patients had aortic stenosis and there were no significant differences between groups. Chronic liver disease, especially with associated portal hypertension, has also been linked to the presence of GI angioectasias and, as reported by some authors, they could be reversed after transjugular intrahepatic portosystemic shunt [34, 38, 39]. We found no significant differences between the two groups concerning the presence of chronic liver disease, which can be partly explained by the small number of patients included. Previous abdominal surgery has also been linked to vascular lesions in the GI tract, such as jejunal varices [40, 41]. However, in our study, we have not found any significant association. We also investigated whether factors known to interfere with hemostasis, such as antithrombotic therapy (antiplatelet or anticoagulant drugs), would be associated with the detection of GI angioectasias. The use of antithrombotic therapy has been associated with a higher diagnostic yield of SBCE, but the possible causal effects with the presence of angioectasias, as well as the risk of recurrent bleeding, are controversial [42, 43]. In our study, the use of antithrombotic therapy was similar between patients in the control group and those with angioectasias.

In conclusion, this study reinforces the importance of evaluating patients' baseline demographic and clinical features before they are selected to undergo SBCE. We confirmed, as reported in previous studies, age and overt OGIB as predictive factors of SB angioectasias. Moreover, we also observed additional associations with some vascular disease risk factors, such as hypertension and hypercholesterolemia. The abovementioned factors should be considered in the process of patients' selection to undergo SBCE. When present, patients should be submitted to SBCE and these factors should be taken into account in the valorization of angioectasias as origin of bleeding. Furthermore, these patients' SBCE recordings may benefit from the use of some new software tools such as flexible spectral imaging color enhancement (FICE), particularly, if the initial study with conventional white light is negative, because it has been shown to increase the detection rate of some SB lesions such angioectasias, as reported in several studies [44–46]. On the other hand, in patients in which all these predictive factors are absent, a more expectant attitude may be followed. It may be acceptable to defer the use of SBCE and insist in the identification of other potential causes of iron deficient anemia (e.g., coeliac disease,* H. pylori* colonization, inflammatory bowel disease, NSAID enteropathy), leaving SBCE as a subsequent possibility. Applying the proposed strategy, the identified predictive factors may be used to increase the diagnostic yield of the SBCE procedure and to reduce the proportion of nondiagnostic examinations.

## Figures and Tables

**Table 1:** Demographic and clinical characteristics of patients.

Characteristic	Control group	Angioectasia group	*P* value
Mean Age (±SD)	53.1 (±18.6)	70.9 (±14.7)	<0.001*
Gender			
Men (%)	18 (34%)	23 (49%)	0.156
Women (%)	35 (66%)	24 (51%)	
SBCE indication			
Occult OGIB (%)	47 (89%)	34 (72%)	0.044*
Overt OGIB (%)	6 (11%)	13 (28%)	
Hypertension			
Yes (%)	24 (45%)	38 (81%)	<0.001*
No (%)	29 (55%)	9 (19%)	
Diabetes mellitus			
Yes (%)	13 (25%)	17 (36%)	0.275
No (%)	40 (75%)	30 (64%)	
Hypercholesterolemia			
Yes (%)	19 (36%)	28 (60%)	0.027*
No (%)	34 (64%)	19 (40%)	
Tobacco use			
Yes (%)	7 (13%)	13 (28%)	0.084
No (%)	46 (87%)	34 (72%)	
Chronic kidney disease			
Yes (%)	8 (15%)	13 (28%)	0.145
No (%)	45 (85%)	34 (72%)	
Chronic liver disease			
Yes (%)	4 (8%)	1 (2%)	0.367
No (%)	49 (92%)	46 (98%)	
Previous abdominal surgery			
Yes (%)	17 (32%)	20 (43%)	0.306
No (%)	36 (68%)	27 (57%)	
Aortic stenosis			
Yes (%)	2 (4%)	5 (11%)	0.249
No (%)	51 (96%)	42 (89%)	
Antiplatelet use			
Yes (%)	15 (28%)	19 (40%)	0.213
No (%)	38 (72%)	28 (60%)	
Anticoagulant use			
Yes (%)	8 (15%)	6 (13%)	0.781
No (%)	45 (85%)	41 (87%)	

SD: standard deviation. *statistically significant.
